# First breeding record of the black‐headed penduline tit (*Remiz macronyx*) in China

**DOI:** 10.1002/ece3.10078

**Published:** 2023-05-20

**Authors:** Hui Wang, Yu Mei, Pinjia Que, Zhengwang Zhang, De Chen

**Affiliations:** ^1^ MOE Key Laboratory for Biodiversity Science and Ecological Engineering, College of Life Sciences Beijing Normal University Beijing China; ^2^ The Specimen Museum of Xinjiang Institute of Ecology and Geography Chinese Academy of Sciences Urumqi China; ^3^ Chengdu Research Base of Giant Panda Breeding Chengdu Sichuan China

**Keywords:** breeding, distribution, Nalati wetland, *Remiz macronyx ssaposhnikowi*, taxonomy

## Abstract

Black‐headed penduline tit (*Remiz macronyx*) is a poorly known bird species mainly distributed in Iran, Turkmenistan, and Kazakhstan. The distribution of black‐headed penduline tit is disjointed and fragmented, and it occurs only along lakes or rivers surrounded by extensive reedbeds. Four subspecies of *R. macronyx* have been recognized (*macronyx*, *neglectus*, *nigricans*, and *ssaposhnikowi*). The *ssaposhnikowi* subspecies was previously known to occur only around lakes in southeastern Kazakhstan. In this study, we reported the first confirmed breeding record of *R. m. ssaposhnikowi* in the Nalati wetland, Ili, Xinjiang, China, extending the distribution range of the black‐headed penduline tit by 350 km to the east. We also obtained new information about the morphology and breeding behavior of *R. m. ssaposhnikowi*, which can be useful for the taxonomy of penduline tits, especially in distinguishing black‐headed penduline tits from Eurasian penduline tits (*R. pendulinus*).

## Graphical abstract

This study is the first to confirm black‐headed penduline tits (Remiz macronyx ssaposhnikowi) breeding in the Nalati wetland, Ili Prefecture, Xinjiang, China. We also obtained new information about the morphology and breeding behavior and photographed their delicate nests for the first time.

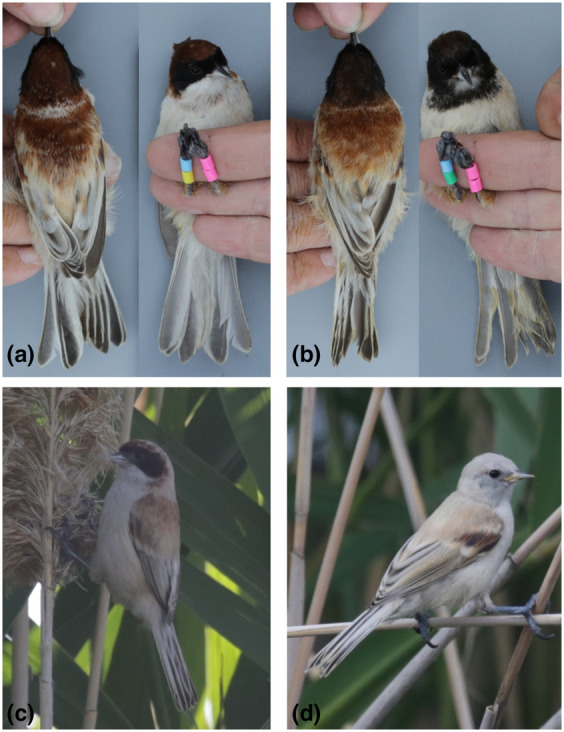

## 1 INTRODUCTION

Penduline tits (*Remiz*) are small passerine species mainly distributed in Europe and Asia, which are famous for their elaborate nests and variable mating and parental care system (Bot et al., 2011; Persson & Öhrström, 1989). The black‐headed penduline tit (*R. macronyx*) is the most variable and least‐known member of this genus. It breeds in low‐lying lakesides and riverbanks with stands of reeds (*Phragmites*) and reedmace (*Typha*) (Madge, 2020). Their nests are different from those of other penduline tit species, which are typically built between two reed stems in a reedbed but sometimes in a tree, depending on what is available (Bot & Van Dijk, 2009; Madge, 2020).

**FIGURE 1:**
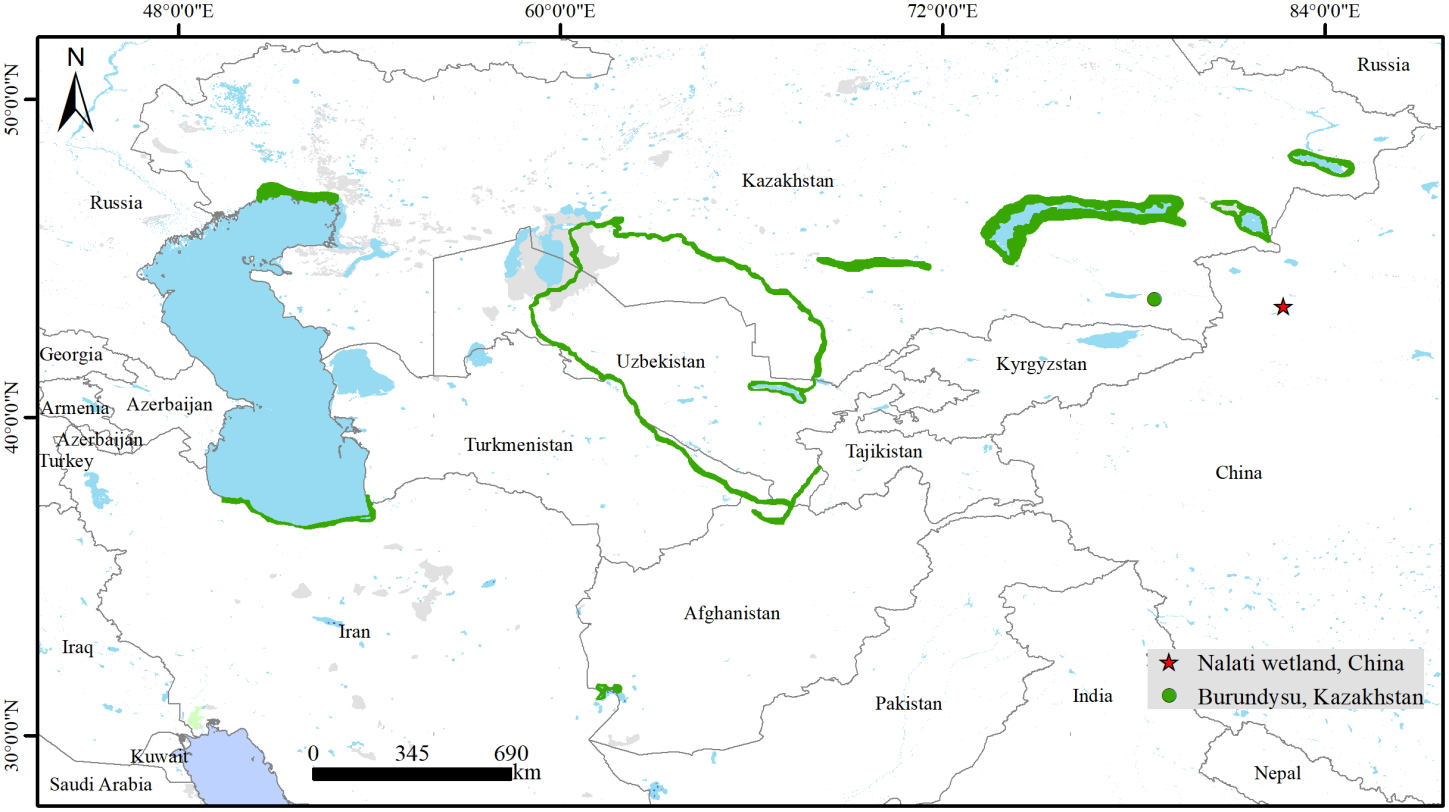
Distribution of the black‐headed penduline tit (*Remiz macronyxv*). The range in green comes from BirdLife International. The red star marks the location of the Nalati wetland, China.

The black‐headed penduline tit is mainly distributed in Iran, Turkmenistan, and Kazakhstan (Figure 1), where four subspecies are recognized, namely, *macronyx*, *neglectus*, *nigricans*, and *ssaposhnikowi* (Bot et al., 2011; Madge, 2020). The nominate subspecies (*macronyx*) occurs locally in reedbeds along rivers and lakes in southwestern Kazakhstan, Uzbekistan, northern and southeastern Turkmenistan, Tajikistan, and probably northeastern Afghanistan (Madge, 2020). The *neglectus* occurs along the southern shore of the Caspian Sea in northern Iran and southwestern Turkmenistan (Barani‐Beiranvand & Aliabadian, 2012; Madge, 2020). *Nigricans* are distributed in southeastern Iran and southwestern Afghanistan, but they are possibly extinct (Barani‐Beiranvand & Aliabadian, 2012). The *ssaposhnikowi* is known to only occur around lakes (Balkhash, Sasykkol, Alakol, and Zaysan) in southeastern Kazakhstan (Bot & Van Dijk, 2009; Gavrilov & Gavrilov, 2005; Madge, 2020). The distribution of the black‐headed penduline tit is disjointed and fragmented, and it only occurs along lakes or rivers surrounded by extensive reedbeds (Figure 1; Madge, 2020). Although some studies suggest that its range includes China (Liu & Chen, 2021; Zheng, 2017), definitive evidence is still lacking (Ma, 2011).

The *ssaposhnikowi* subspecies is rare and variable in morphology, whether it should be recognized as a separate subspecies is controversial (Bot & Van Dijk, 2009; Svensson & Shirihai, 2018; Vaurie, 1957). To date, most records of this subspecies are found in the Ili River basin, and the easternmost known distribution in the Ili River basin is Burundysu (Figure 1; 43°42′N 78°39′E), Almaty Province, Kazakhstan (Gavrilov & Gavrilov, 2005). We speculated that this subspecies may occur in Ili, Xinjiang, China, where the Ili River originates and a similar habitat occurs. In this study, we conducted several field trips upstream of the Ili River in search of the black‐headed penduline tit, and this species was discovered in China in 2022. We also acquired valuable data about its morphology, breeding behavior, and taxonomy during the field expeditions.

## 2 METHODS AND RESULTS

### 2.1 The record site, habitat, and population size

From May to July 2022, black‐headed penduline tits were found in the reedbeds in the Nalati wetland, Xinyuan County, Ili Prefecture, Xinjiang, China (Figure 1). The Nalati wetland is located along the Künes River, which merges with the Tekes River and other rivers to form the Ili River.

**FIGURE 2:**
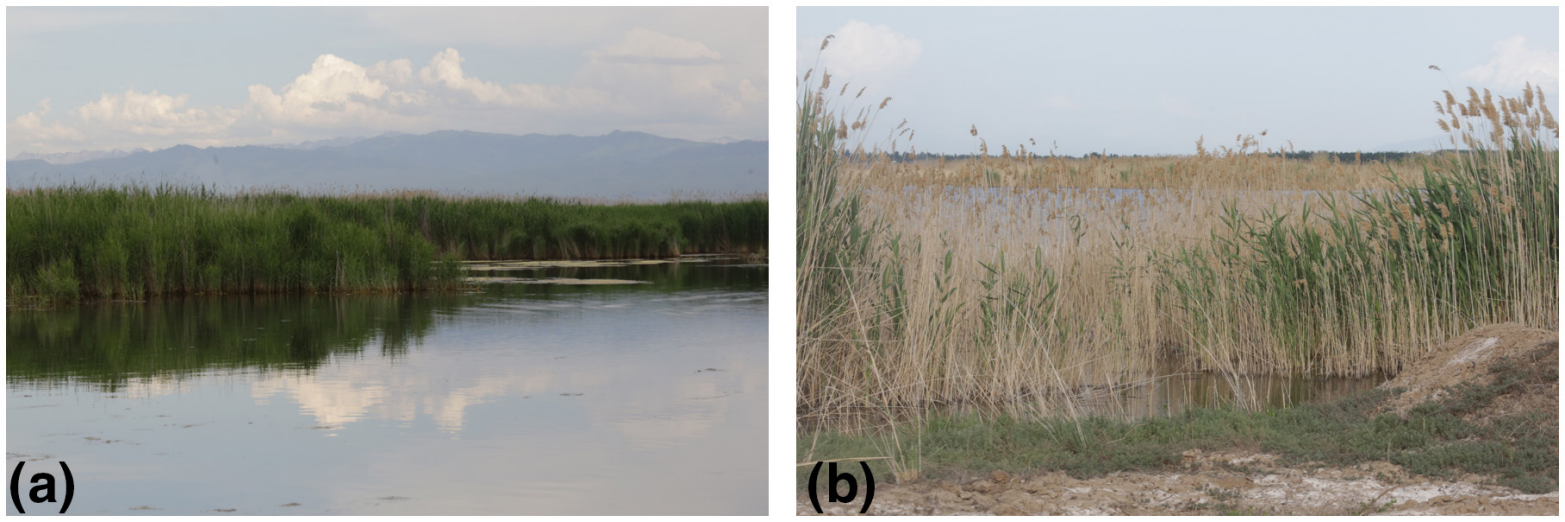
Typical habitat of black‐headed penduline tit (*Remiz macronyx ssaposhnikowi*). (a) Nalati National Wetland Park, China, May 2022. (b) Zhongyangchang Lake, China, May 2022.

The area of the Nalati wetlands is very large, including the Nalati National Wetland Park (43°30′N 82°42′E) and several lakes around it. The Nalati National Wetland Park has a total planned area of 140.52 km^2^, of which 139.45 km^2^ are wetlands with landscapes such as rivers, swamps, lakes, and meadows. There are extensive reeds (*Phragmites australis*) that are suitable for the breeding of black‐headed penduline tits (Figure 2a). Birds in Nalati National Wetland Park seem quite common; we surveyed a small area (less than 1.5 km) of the park by tourist boat and saw more than 20 birds in 30 min. In addition, another 11 birds (eight males, one female, and two juveniles) were found in a small lake (named Zhongyangchang Lake, 0.4 km^2^ in size, 43°33′N 82°43′E) 3 km north of the park, which is surrounded by sparse reeds (Figure 2b).

### 2.2 Appearance and measurements

We caught five males in Zhongyangchang Lake by a mist net (6 m × 3 m) setting close to the reeds. A playback of black‐headed penduline tit call (downloaded from https://xeno‐canto.org/) was used to attract the birds. All trapped birds were marked by four color rings and photographed in standard manners with a camera (Canon 5D Mark III with a 50 mm lens). Six morphometric traits (weight, bill length, tarsus length, wing length, tail length, and body length) were measured before the bird was released in the field. The morphometric traits of the five males are listed in Table 1.

**TABLE 1:** Measurements of five *Remiz macronyx ssaposhnikowi* males trapped during the research period; one individual did not have its tail length and body length measured due to molting.

Morphometric characters	Mean (range)
Weight	9.58 (9.26–10.10)
Bill length (culmen)	10.89 (10.70–11.52)
Tarsus length	14.92 (14.60–15.32)
Wing length	58.50 (57.50–60.00)
Tail length	55.88 (54.5.00–57.50)
Body length	123.5 (121.00–125.00)

*Note*: Length measurements in millimeter (mm), weight in gram (g).

**FIGURE 3:**
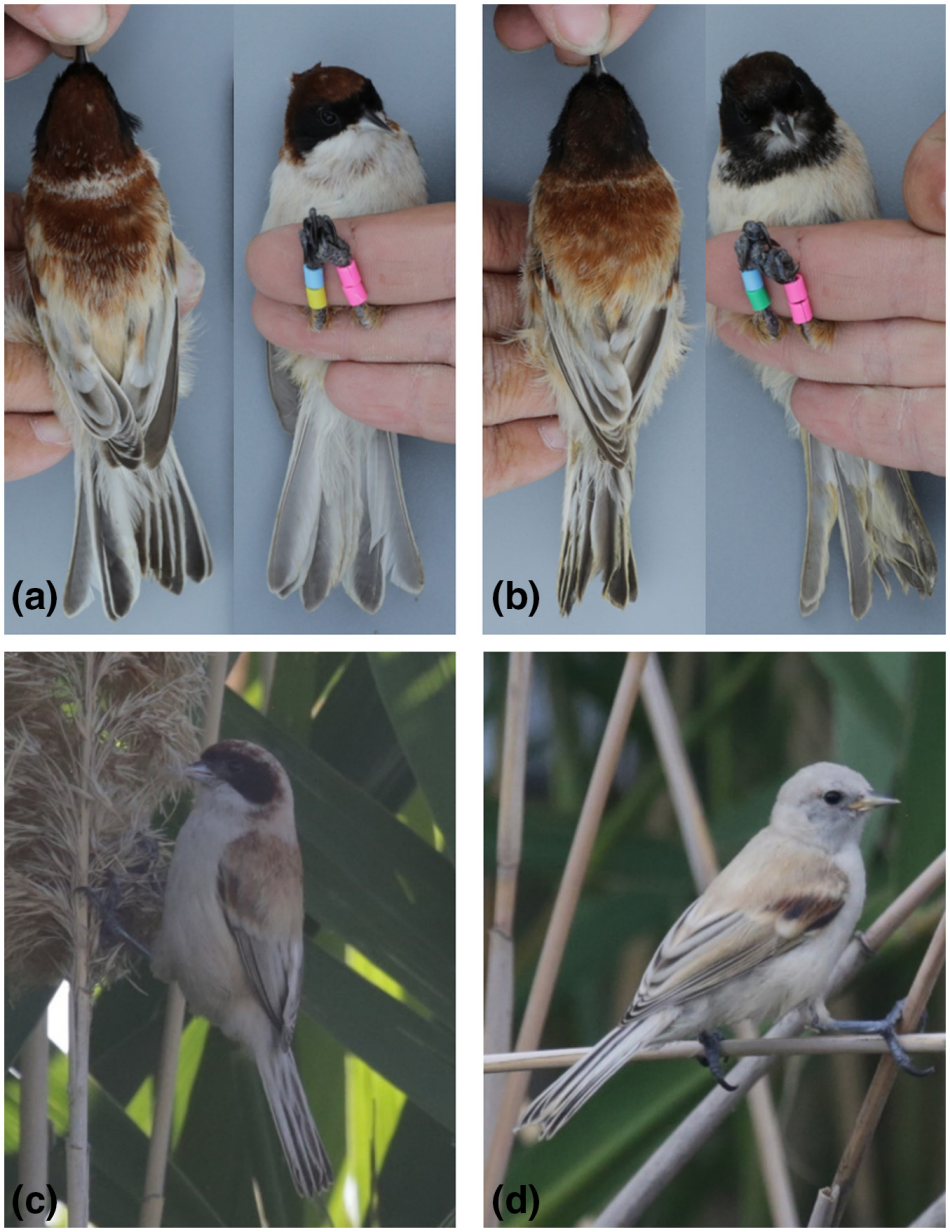
Photos of the black‐headed penduline tit (*Remiz macronyx ssaposhnikowi*). (a) Typical male; (b) the only observed male with a sooty black throat and upper breast; (c) female; (d) juvenile.

All the males had a black mask, a chestnut‐brown crown, and nape, and most of them had a whitish throat (Figure 3a), except one who had a sooty black throat and upper breast (Figure 3b). The mantle and scapulars were deeply dark red‐brown, and the flight feathers had broad white fringes, creating a white wing panel; the underparts were buff. Female is similar to male but less well marked and has a wide black mask, a clear red‐brown fringe on the head above the forehead patch extending to both sides of the crown. However, the red‐brown area in the head is smaller in females than in males, and the mantle and scapula are paler in females than in males (Figure 3c). On 28 July, the last time we visited the lake, we saw two juveniles foraging in the reedbeds together. They have large feet like the adults, but the head and upper parts are dull grayish, and the underparts are light buff (Figure 3d). Given these morphological features and its close distribution to the black‐headed penduline tits (Figure 1), the birds we found in the Nalati wetland are supposed to be the *ssaposhnikowi* subspecies of black‐headed penduline tits.

### 2.3 Nests and breeding behavior

**FIGURE 4:**
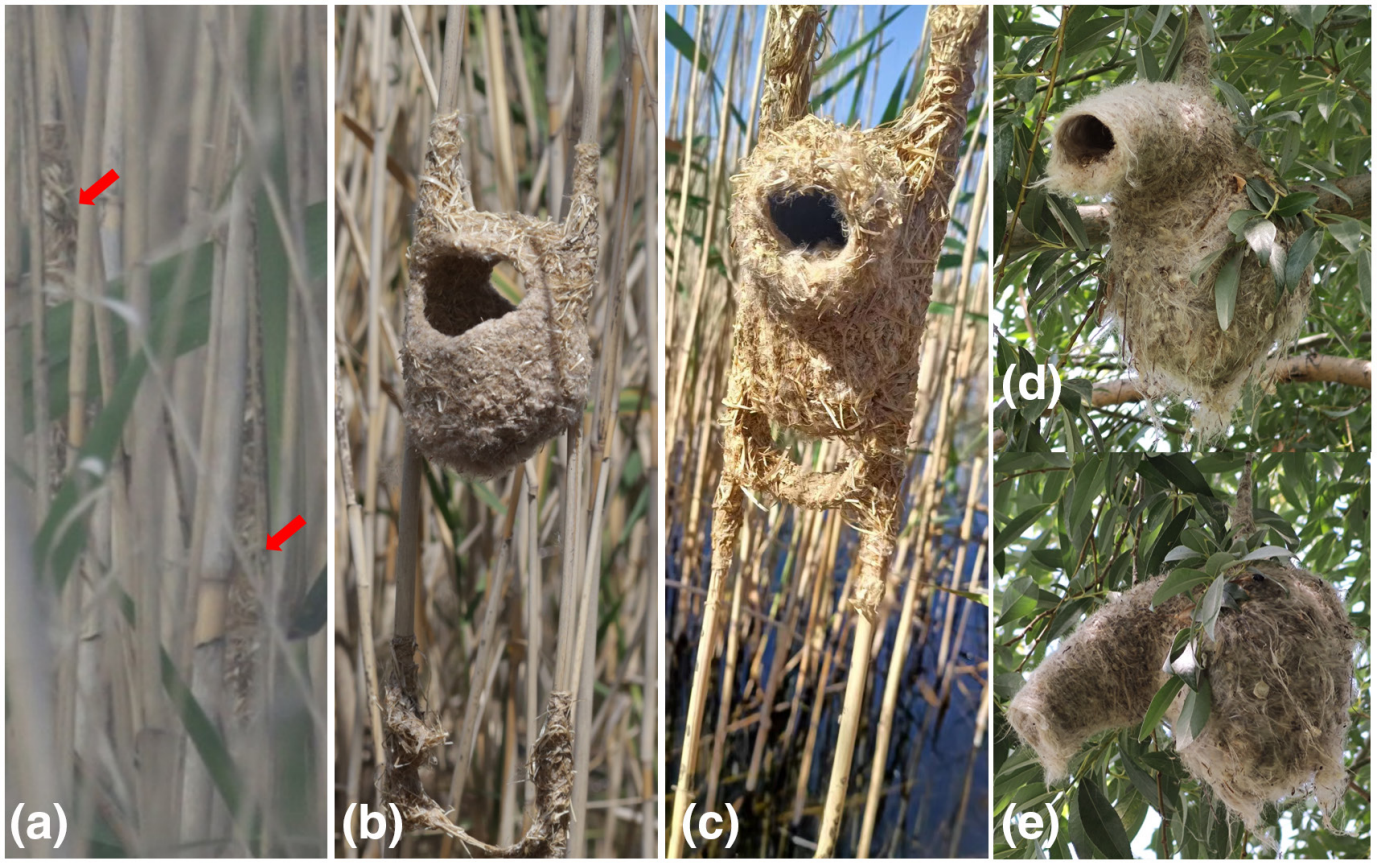
Nests of penduline tits. (a–c) Nests of black‐headed penduline tit (*Remiz macronyx ssaposhnikowi*) in different stages. (a) Stage A, a few plant fibers were wrapped around each of the two adjacent reeds (marked with red arrows); (b) Stage D, shaped as a “basket”; (c) Stage F, a complete globular pouch with an entrance tube at the side. (d,e) Nests of white‐crowned penduline tit (*R. coronatus*) in (d) front view and (e) side view, stage F.

We found four nests in Zhongyangchang Lake. The minimum distance between two nests was approximately 200 m. All nests were built between two reed stems with reed flowerheads and other plant fibers, placed approximately 50–100 cm above water (Figure 4). A nest at an early stage was found on May 14, 2022, and a few plant fibers were wrapped around each of the two adjacent reeds (Figure 4a; defined as stage A). Like all other penduline tits, only a single male was building the nest during this stage. On 9 June, the A‐stage nest had completely developed (stage F). The nest has an elongated globular pouch with an entrance tube at the side, and a connecting braid is built below the nest (Figure 4c). There were six white eggs in the nest, all of which were under incubation. We monitored the nest for approximately 1 h, and the incubation was carried out by females alone. The other three nests, shaped as a “basket” (stage D, Figure 4b), were attended by a single male; perhaps the males were still unmated on June 9, 2022.

## 3 DISCUSSION

This study is the first to confirm *R. m. ssaposhnikowi* breeding in the Nalati wetland, Ili Prefecture, Xinjiang, China, which is 350 km east of the known easternmost distribution along the Ili River basin in Burundysu, Kazakhstan (Figure 1). Thus, our findings extended the eastern distribution range of black‐headed penduline tits. Black‐headed penduline tits in Nalati National Wetland Park seem common, with more than 20 birds in an informal 1.5 km boat survey, where more than 100 km^2^ reedbeds are in this wetland. By contrast, Bot and Van Dijk (2009) reported that the population of *R. m. ssaposhnikowi* in Topar Lakes appeared to be of low density, and only 14 birds were found. The reedbeds in Topar Lakes are sparse and discontinuous, similar to those in Zhongyangchang Lake (Figure 2b). Overall, *R. m. ssaposhnikowi* prefers extensive reedbeds, and it can also breed in lakes or rivers with sparse reedbeds.

Penduline tits are famous for their delicate nests. All species in genus *Remiz* built a domed nest with a tube at the side (Figure 4). Nests can be divided into three types based on their location and shape: building a nest in a tree (tree nest, Figure 4d,e), building a nest between two reed stems (reed nest, Figure 4a–c), and building a tree nest‐like nest between two reed stems (transitional nest) (Burckhardt, 1948). There are several differences between the reed nest and tree nest. First, the reed nest was shaped like a cylinder, whereas the tree nest was shaped like a pear. Second, the reed nests are built between two parallel reed stems “tied” together with plaited strips of plant fibers for extra support, and the left and right sides of the nest are connected to reeds with plant fibers (Figure 4a–c); the tree nest hangs in tree branches only with some plant fibers connected on the top (Figure 4d,e). Finally, the tube of the entrance is shorter in the reed nest, and the nest materials used in the reed nest are coarser than those in the tree nest (Figure 4). Most penduline tits build their nests in trees (Bot et al., 2011). Previous studies have described the nest structure and divided the nest progress into different stages from A to F (Van Dijk, Szentirmai, & Székely, 2007; Zheng et al., 2018), and there are also numerous photographic records online. However, descriptions and photographic records of the reed nest are quite rare. In this study, we photographed reed nests at three stages for the first time (Figure 4a–c) and describe the differences between different nest types.

Another attractive feature of penduline tits is their variable parental care system. The Eurasian penduline tit (*R. pendulinus*) consistently conducts uniparental care within and across populations. Either males, females, or both may desert the clutch during the egg‐laying stage (Pogány et al., 2008; Van Dijk, Szentirmai, Komdeur, & Székely, 2007). Biparental care is thought to be the most common strategy for white‐crowned penduline tit (*R. coronatus*), accounting for 78% of all breeding nests (Ball et al., 2017). In the Chinese penduline tit (*R. consobrinus*), uniparental care was the dominant pattern, accounting for 80% of nests, female‐only care was performed in 71% of nests, and male‐only care was performed in 9% of nests (Zheng et al., 2018). However, little information is available for the black‐headed penduline tit. We have been able to study only one breeding case of *R. m. ssaposhnikowi*, and only females performed the parental task during incubation. Another record of the parental care of the black‐headed penduline tit also comes from *R. m. ssaposhnikowi*; the male abandoned the nest during the egg‐laying stage and left the female to carry over the rest of the work (Bot & Van Dijk, 2009). Based on these two observation cases, female‐only care seems to be the most common parental care strategy in *R. m. ssaposhnikowi*, although more observations are needed.

The *ssaposhnikowi* subspecies of the black‐headed penduline tit was described by Herman Johansen in 1907, and it was recognized as a valid subspecies by Portenko (1955), although many authors do not recognize it, due to its rather variable morphology (Bot & Van Dijk, 2009; Svensson & Shirihai, 2018; Vaurie, 1957). The population in Lake Balkhash and Topar Lakes exhibits an extreme degree of individual variability, and the head patterns of some individuals resembled the Eurasian penduline tit *R. p. caspius*, with chestnut‐brown on the crown and nape, while others are identical to the black‐headed penduline tit *R. m. macronyx*, with all black head (Bot & Van Dijk, 2009; Vaurie, 1957). Bot and Van Dijk (2009) suspected that *R. m. macronyx* and *R. p. caspius* may coexist in that area, and *R. m. ssaposhnikowi* may be identical to *R. p. caspius*. However, our observations in Nalati do not support this view. First, the measurements of morphometric characters are quite different between *R. m. ssaposhnikowi* in the Nalati wetland and *R. p. caspius*. The tarsus length (14.9 mm vs. 14.3 mm) and tail length (55.88 mm vs. 47.3 mm) of *R. m. ssaposhnikowi* in Nalati are larger than those of *R. p. caspius* (morphometric data were from Svensson & Shirihai, 2018; statistical comparison was not performed because they only provide the mean value and range). The toes and claws of *R. m. ssaposhnikowi* are also very strong (see Figure 3d). Furthermore, none of the birds we saw in Nalati had an entire black head like *R. m. macronyx*. There was one bird whose throat and upper breast were black (Figure 3b), but the morphometric characteristics and behavior were not different from those of the other groups. In addition, all *R. m. ssaposhnikowi* in Nalati built reed nests, whereas *R. p. caspius* always built tree nests. Overall, the *ssaposhnikowi* in Nalati is morphologically and behaviorally different from *R. p. caspius* and has plumage characteristics unique to other black‐headed penduline tits subspecies. It should be treated as a separate subspecies of the black‐headed penduline tit.

The taxonomy of the black‐headed penduline tit is still controversial. Previous research has combined all penduline tits into one wide‐ranging polytypic species, *Remiz pendulinus*, while others have divided it into four species (Eck & Martens, 2006; Harrap, 1996; Madge, 2008). Barani‐Beiranvand et al. (2017) investigated the phylogenetic relationship within *Remiz* using mitochondrial and microsatellite genotyping, which showed that *R. coronatus* and *R. consobrinus* are genetically well differentiated, but there was no evidence for significant differentiation between *R. pendulinus* and *R. macronyx* (Barani‐Beiranvand et al., 2017). Therefore, the main riddle has been focused on the phylogenetic relationship between the Eurasian penduline tit and black‐headed penduline tit, and whether they should be treated as different species is a difficult question. Our study provided some new data about this taxonomic puzzle. According to our observations, the black‐headed penduline tit in Nalati differs from the Eurasian penduline tit in morphology, habitat preference, and nest type. The black‐headed penduline tit in Nalati can be easily distinguished from the other subspecies of Eurasian penduline tit except *caspius* based on the chestnut‐brown crown and nape. Although there are no diagnostic plumage differences to distinguish *R. p. caspius* and *R. m. ssaposhnikowi* (Vaurie, 1952; Vaurie & Koelz, 1950), *R. m. ssaposhnikowi* in Nalati has a relatively longer tail and larger claws than *R. p. caspius* (see Discussion above). Larger biometric measurements were also found in other populations of black‐headed penduline tits, for example, *R. m. ssaposhnikowi* in the Topar Lakes (Bot et al., 2011), *R. m. macronyx* in Turkey (Kittelberger et al., 2022) and Transcaspia (Barani‐Beiranvand & Aliabadian, 2012), which are possibly adaptations to living in reedbeds. Furthermore, most Eurasian penduline tits build their nests in trees (including *R. p. caspius*), while black‐headed penduline tits usually inhabit reedbeds and build their nests between two reed stems. In summary, the black‐headed penduline tits and Eurasian penduline tits are morphologically and behaviorally distinct. However, due to the hybridization between some subspecies in nature, it is difficult to conclude that they are separate species based on these limited observations (Barani‐Beiranvand & Aliabadian, 2012; Vaurie, 1957). It is hoped that our observations will prompt researchers to conduct more in‐depth studies of this group, including high‐throughput genome‐scale sequencing data in combination with phenotypic data, which will help to determine the hybridization and evolutionary relationships between these populations.

Black‐headed penduline tit is one of the most poorly known bird species, with only 60 records worldwide on eBird (eBird, 2023). Recent studies have reported the westernmost record of the black‐headed penduline tit in Turkey (Kittelberger et al., 2022), and our study extends the easternmost distribution of this species in the Ili River basin, which implies that our current knowledge of the distribution of the black‐headed penduline tit is still limited. We recommend that other field researchers or bird watchers search for or pay more attention to this species in future fieldwork.

## AUTHOR CONTRIBUTIONS

**Hui Wang:** Formal analysis (lead); investigation (lead); writing – original draft (lead). **Yu Mei:** Investigation (supporting); resources (supporting); writing – review and editing (supporting). **Pinjia Que:** Investigation (supporting); writing – review and editing (supporting). **Zhengwang Zhang:** Conceptualization (supporting); funding acquisition (supporting); supervision (supporting); writing – review and editing (supporting). **De Chen:** Conceptualization (lead); funding acquisition (lead); investigation (supporting); project administration (lead); supervision (lead); writing – original draft (supporting); writing – review and editing (supporting).

## Data Availability

Data sharing not applicable to this article as no datasets were analysed during the current study.
